# Simultaneous Detection of Bluetongue Virus Serotypes Using xMAP Technology

**DOI:** 10.3390/microorganisms8101564

**Published:** 2020-10-11

**Authors:** Martin Ashby, Paulina Rajko-Nenow, Carrie Batten, John Flannery

**Affiliations:** The Pirbright Institute, Ash Road, Pirbright, Woking, Surrey GU24 0NF, UK; paulina.rajko-nenow@pirbright.ac.uk (P.R.-N.); Carrie.batten@pirbright.ac.uk (C.B.); john.flannery@pirbright.ac.uk (J.F.)

**Keywords:** Bluetongue virus, serotyping, multiplexing, xMAP, diagnostics, RT-qPCR, epidemiology

## Abstract

Bluetongue is an economically important disease of ruminants caused by the bluetongue virus (BTV). BTV is serologically diverse, which complicates vaccination strategies. Rapid identification of the causative BTV serotypes is critical, however, real-time PCR (RT-qPCR) can be costly and time consuming to perform when the circulating serotypes are unknown. The Luminex xMAP technology is a high-throughput platform that uses fluorescent beads to detect multiple targets simultaneously. We utilized existing BTV serotyping RT-qPCR assays for BTV-1 to BTV-24 and adapted them for use with the xMAP platform. The xMAP assay specifically detected all 24 BTV serotypes when testing reference strains. In all BTV-positive samples, the sensitivity of the BTV xMAP was 87.55% whereas the sensitivity of the serotype-specific RT-qPCR was 79.85%. The BTV xMAP assay allowed for the specific detection of BTV serotypes 1–24 at a lower cost than current RT-qPCR assays. Overall, the assay provides a useful novel diagnostic tool, particularly when analyzing large sample sets. The use of the BTV xMAP assay will allow for the rapid assessment of BTV epidemiology and may inform decision-making related to control and prevention measures.

## 1. Introduction

Bluetongue (BT) is a world Organization for Animal Health (OIE) notifiable disease of ruminants. Acute clinical disease is mainly seen in sheep, with cattle thought to be the reservoir host; additionally, goats and some deer species can be infected [1,2]. The disease is caused by the BT virus (BTV) and is primarily transmitted by adult *Culicoides* midges [3]. Clinical signs can include fever, depression, lameness, oedema of the lips, tongue, and head, conjunctivitis, coronitis, nasal discharge, hyperemia, and death. Animals that recover from BT can show prolonged convalescence and may exhibit reduced milk yield or reduced weight-gain and wool break. The BTV genome consists of ten segments of linear double-stranded RNA that encode for a number of structural (VP1-VP7) and non-structural (NS1-NS5 and NS3a) viral proteins [4,5]. The structural protein VP2, encoded by segment 2, shows the highest degree of variation and is the main determinant of BTV serotype [6]. Until 2008, 24 BTV serotypes had been detected based on interactions between VP2 and neutralizing antibodies in the ruminant host. However, since that time, a number of additional BTV serotypes (of reduced virulence) have been detected, which include serotypes 25 to 29 [7,8,9,10], in addition to the as-yet unclassified BTV strains TUN2017, BTV-X ITL2015, V196/XJ/2014, and SPvvvv/02 [11,12,13,14].

The most appropriate prevention and control strategy for BT involves vaccination of susceptible hosts against the circulating BTV serotype(s). At present, RT-qPCR assays targeted towards the individual BTV serotypes provide the most useful means of doing so. However, in areas where multiple serotypes circulate or there is an incursion of serotypes new to the region, RT-qPCR assays can be time consuming and costly to perform. In the EU, animal movement restrictions are imposed in an effort to prevent further spread of BTV (with or without concurrent vaccination programs) [15,16]. As of April 2020, six BTV serotypes (BTV-1, BTV-2, BTV-3, BTV-4, BTV-8, and BTV-16) are circulating in the EU and have necessitated animal movement restriction zones in 14 Member States [17]. In the absence of relevant epidemiological data, the identification of BTV serotype can be hindered by the deductive process required to rule out the many potential BTV serotypes circulating. For instance, the detection of BTV-8 in Switzerland and Germany in 2017 and 2018, respectively, would have required the exclusion of BTV-4 since both BTV-4 and BTV-8 were already circulating in France at the time. The two main corridors of BTV incursion from Africa (through the eastern and western Mediterranean routes) into the EU [18,19] are an ever-present risk for the incursion of additional BTV serotypes. BTV-3 first incurred in the EU in 2017 in Italy, however, BTV-3 had already been detected a year prior in Tunisia, which may have helped expedite the identification [20]. However, previous detection of BTV-6, BTV-11, BTV-14 in the EU were not epidemiologically traced to neighboring countries but rather to the use of modified live vaccines [21,22,23]. Thus, knowledge of the circulating serotypes in North African countries allows for timely risk assessments to be conducted. Given the numerous BTV serotypes identified thus far in the EU, multiplexing of RT-qPCR assays offers limited benefit due to the constraints of the instrumentation currently available, which allows for triplex or tetraplex assays to detect three or four serotypes at most. Indeed, the use of single or multiplex RT-qPCR serotype specific assays is not cost or time-effective when analyzing sample sets from areas where multiple serotypes co-circulate.

The Luminex^®^ xMAP technology (where x is the unknown analyte and MAP stands for Multi-Analyte Profiling) has the potential to allow for the detection of numerous individual targets in a single reaction [24]. The Luminex technology was originally developed for the detection of multiple analytes, such as cytokines and B-cells [25], however, xMAP multiplex technology has evolved to provide a diagnostic tool in clinical and veterinary fields. xMAP assays have been used in the detection of both proteins [26,27] and nucleic acid [28,29]. In the latter, a PCR step is initially performed to amplify and label the target sequence, which is then hybridized with a polystyrene microsphere via a coupled complimentary oligo. Microsphere sets contain unique ratios of two fluorescent dyes, which make them spectrally distinct from one another. Detection of the microsphere and hybridized oligo using a Luminex instrument provides semi-quantitative information on the presence of target material in the sample. xMAP assays have been developed for the detection of rotaviruses, noroviruses, sapoviruses, astroviruses, and adenoviruses in clinical [30] and environmental samples [31]. In the veterinary diagnostics field, Chen et al. [32] developed an xMAP assay for porcine respiratory syndrome virus, porcine circovirus type 2, porcine pseudorabies virus, classical swine fever virus, and porcine parvovirus. In all studies, comparable specificity to qPCR-based assays was achieved and results were generated with a quicker turnaround time. The xMAP technology has since been commercialized in the xTAG^®^ Gastrointestinal Pathogen Panel (for norovirus, rotavirus, and adenovirus), which has been used successfully in clinical settings [33].

The true impact of the numerous BTV serotypes to ruminant health worldwide has not been determined, partly due to the significant costs associated with serotyping using RT-qPCR assays. We aimed to determine whether the xMAP technology could be applied to BTV serotyping and thus provide for an appropriate high-throughput diagnostic platform. Herein we describe the development of an xMAP assay for the detection of BTV serotypes 1–24, which are the only serotypes that currently require OIE notification. The assay was developed using previously published primer and probe sequences [34]. Following optimization, we performed an evaluation of the BTV xMAP assay alongside serotype-specific RT-qPCR assays.

## 2. Materials and Methods

### 2.1. RNA Extraction

Extraction of RNA from EDTA blood and tissue culture supernatant was performed using the KingFisher Flex automated extraction platform (ThermoFisher Scientific, Paisley, UK) and the MagVet Universal nucleic extraction kit (ThermoFisher, Paisley, UK). The sample volume was 100 µL and RNA was eluted into 80 µL of elution buffer. RNA was stored at −20°C until use.

### 2.2. BTV Serotyping Primers for RT-PCR Stage of BTV xMAP Assay

Previously published primers [34] were used in the RT-PCR step of the BTV xMAP assay, one exception was the BTV-24 forward primer, which was lengthened by 3 nucleotides to increase the primer melting temperature (Table S1). All primers were designed to amplify serotype-specific regions within segment 2 of the BTV genome. Primers were modified to include a biotin moiety at the 5′ end of the forward or reverse primer and were purchased from ThermoFisher. For the majority of primer pairs, an asymmetric PCR approach was used with a 1:4 ratio of non-biotinylated: biotinylated primer. However, for some serotypes, a 1:4 ratio caused sub-optimal amplification and the primer ratios for these serotypes were individually optimized to improve assay performance. Primers for BTV serotypes 1 to 24, three of which contained two topotypes (BTV-2, BTV-16, and BTV-9), were split into either 3 serotyping panels (Table S2 supplementary data) each consisting of primers for 8–9 serotypes, or 5 serotyping panels each consisting of primers for 5 serotypes (Table S3). Primers from each panel were diluted to the required working concentration in nuclease-free water.

### 2.3. RT-PCR

Two µL from one of the xMAP serotyping panel primer mixes was added to 5 µL extracted BTV RNA and denatured at 95 °C for 5 min. Following denaturation, a PCR reaction mix containing: 12.5 µL express qPCR supermix (ThermoFisher, Paisley, UK), 2.5 µL express superscript mix (ThermoFisher, Paisley, UK), and 3 µL nuclease-free water was added to each sample, equating to a total volume of 25 µL. Cycling conditions were 50 °C for 15 min, 95 °C for 2 min, and then 40 cycles of 95 °C for 15 s, 56 °C for 30 s, and 68 °C for 1 min.

### 2.4. Coupling of Microsphere to Capture Probes

Capture probes used in the study were based on previously published RT-qPCR probes [34] (Table S1) but were modified to include a 5′ amine attached via a C12 linker (Eurofins genomics, Ebersberg Germany). Luminex Magplex microsphere sets 12 to 22 (Luminex Corporation, Hertogenbosch Netherlands) were vortexed and sonicated for 20 s before 5 × 10^6^ microspheres were transferred to an Eppendorf LoBind microcentrifuge tube. Coupling of capture probe was performed in accordance with the manufacturer’s recommendations [24]. Microspheres were resuspended in 50 µL 0.1 M 4-Morpholineethanesulfonic acid (MES hydrate) pH 4.5 (Sigma Aldrich, Dorset, UK). Two µL of capture probe (100 µM) was added to the microspheres along with 2.5 µL 10 mg/mL 1,3-Propanediamine,N-(ethylcarbonimidoyl)-N,N-dimethyl-, monohydrochloride (EDC) (ThermoFisher), the suspension was then vortexed before being incubated in the dark at room temperature. After 30 min incubation, another 2.5 µL 10 mg/mL EDC was added to the solution, which was then incubated for a further 30 min in the dark at room temperature. One mL 0.02% tween-20 (Sigma-Aldrich) was added to each tube before removing the supernatant through magnetic separation of the microspheres in a magnetic rack. Microspheres were then washed once with 1 mL 0.1% SDS (Sigma-Aldrich), the supernatant was removed, and microspheres were resuspended in 500 µL Tris-EDTA buffer pH 8.0. Microspheres were stored in the dark at 5 ± 3 °C until use.

### 2.5. Detection of BTV Serotypes Using xMAP Assay

Microspheres with coupled capture probes were diluted in 1.5X TMAC solution (4.5 M Tetramethylammonium chloride, 0.15% N-lauroylsarcosine sodium salt solution, 75 mM Tris, 6 mM EDTA) to a concentration of 76 microspheres/µL. The diluted microspheres were grouped according to the 5-plex panels outlined in Supplementary Table S3 and the suspension was vortexed for 20 s with 33 µL of the resulting mix added to the designated wells of a 96-well PCR plate. To each well of a 96-well micro plate, 5 µL PCR product from the corresponding serotype panel was added along with 12 µL TE buffer. The suspension was thoroughly mixed before being heated in a thermocycler for 90 s at 95°C and then 20 min at 55 °C. Twenty-five µL Streptavidin-R-Phycoerythrin (SAPE) (10 µg/mL) was added to each well and the plate was incubated for 20 min at 55 °C.

### 2.6. BTV xMAP Data Collection and Analysis

The instrument used was a Bio-Plex 200 instrument (Luminex corp., Ebersberg, Germany) and data collection was performed using the Luminex xPONENT 3.1 software. The software has pre-defined detection gates for each microsphere set based on the fluorescence of two impregnated fluorescent dyes. The fluorescence emitted from bound SAPE was also detected and for each microsphere set, a median fluorescence intensity (MFI) was calculated within the software based on a minimum of 500 detectable microspheres. At least two negative controls were included for each batch of samples analyzed on the instrument, and the mean MFI of the negative controls was subtracted from the MFI obtained for each sample. Throughout the initial assay development stages, the background fluorescence did not exceed an MFI of 150, therefore, this was chosen as the minimum threshold for which a sample was considered positive.

### 2.7. BTV Serotype-Specific RT-qPCR

BTV serotype-specific RT-qPCR was performed to confirm results obtained using the BTV xMAP assay. The RT-qPCR used serotype-specific primers and probes [34] targeting segment 2 of the BTV genome. A reaction mix was prepared using 10 µL EXPRESS SuperMix Universal (ThermoFisher, Paisley, UK) 400 nM forward and reverse primers, 100 nM probe, 0.4 µL ROX, 2 µL EXPRESS SuperScript Mix (ThermoFisher, Paisley, UK), and molecular biology grade water to achieve a final volume of 17 µL per well. Three µL template RNA (previously denatured at 95 °C for 5 min) was added to a designated well and RT-qPCR was performed on an Applied Biosystems 7500 Fast real-time PCR system (ThermoFisher, Paisley, UK) using the following cycle conditions: 50 °C for 15 min, 95 °C for 20 s, followed by 45 cycles of 95 °C for 3 s, and 60 °C for 30 s. Samples were considered positive if C_T_ values were ≤38.

### 2.8. BTV Group-Specific RT-qPCR 

A BTV group-specific RT-qPCR (targeting segment 10) [35] was prepared using the same volumes as described for the BTV serotype-specific RT-qPCR (above). Template RNA (5 µL, previously denatured at 95 °C for 5 min) was added to a designated well and RT-qPCR was performed on an Applied Biosystems 7500 Fast real-time PCR system (ThermoFisher, Paisley, UK) using the following cycle conditions: 50 °C for 15 min, 95 °C for 20 s followed by 45 cycles of 95 °C for 3 s, 56°C for 30 s and 72 °C for 30 s. Samples were considered positive if C_T_ values were ≤38.

### 2.9. Assay Optimization

The ratio of biotinylated primer to non-biotinylated primer, the annealing temperature used in the xMAP PCR, the temperature of capture probe hybridization with biotinylated target, and the duration of the SAPE-biotin incubation were all investigated to identify the optimal experimental conditions. Due to the number of serotypes incorporated (*n* = 24) within the assay, it was not practical to test each different condition for each different serotype. Instead, a subset of serotypes was assessed, and the optimal conditions identified were applied to all other serotypes. Any serotype that was poorly detected once the optimal conditions had been applied was then individually assessed and further optimized. All optimization was performed in a multiplex format apart from the optimization of primer ratios, which was performed as a series of singleplex reactions.

The initial design of the serotyping panels was based on minimizing the variation in melting temperatures of primers within each panel. Modifications were made to address the inhibitory effects that occurred when some primers were combined in a single mix. A detailed analysis of BTV-24 amplification inhibition was undertaken by performing several RT-qPCR duplex assays utilizing BTV-24 primers and primers from other serotypes. Inhibition of BTV-24 amplification was calculated by the following equation:

$$100-\left(\frac{\;\Delta \mathrm{flourescence}\;\mathrm{BTV}-24\;\mathrm{duplex}\;}{\Delta \mathrm{fluorescence}\;\mathrm{BTV}-24\;\sin\mathrm{gleplex}\;}\times 100\right)$$

Only the BTV-24 probe was included in the reaction and the primer and probe sequences used in this assessment were the same as those used in the Luminex xMAP assay. 

### 2.10. Panel of Samples for Evaluation of BTV xMAP

An assessment of the BTV xMAP assay was performed using 103 samples. This panel was comprised of 32 bovine (*n* = 28), ovine (*n* = 1), and caprine (*n* = 3) EDTA blood samples that had been submitted to The Pirbright Institute, UK for diagnosis. In addition, 47 EDTA blood samples of bovine (*n* = 14) and ovine (*n* = 33) origin from the European Union BTV proficiency testing schemes 2016–2019 and 24 BTV reference strains (cell culture supernatant representing BTV serotypes 1–24) from the Orbivirus Reference Collection, Pirbright and available through the European Virus Archive goes Global (EVAg) consortium (https://www.european-virus-archive.com/).

### 2.11. Limit of Detection

The limit of detection of the serotype-specific RT-qPCR assays was previously determined [34], nevertheless, an assessment of the limit of detection of the xMAP was performed using three randomly-selected BTV isolates: BTV-3, BTV-15, and BTV-22. Cell cultures of these BTV serotypes were diluted in phosphate buffered saline (PBS) from 10^−1^ to 10^−6^ and RNA was extracted using the KingFisher flex extraction platform. The eluted RNA was analyzed in triplicate using both the BTV xMAP assay and the serotype-specific RT-qPCR.

### 2.12. Repeatability of BTV xMAP

To determine intra-assay repeatability of the BTV xMAP assay, RNA for BTV serotypes 1, 4, 7, and 8 was analyzed in triplicate on a single plate and the % coefficient of variation (%CV) was calculated. To determine inter-assay repeatability, RNA for BTV serotypes 1, 4, 6, and 8 was analyzed in triplicate across three separate runs and the % CV was calculated.

### 2.13. Price Comparison of BTV xMAP with Serotype-Specific RT-PCR Assays

Reagent and consumable costs used in the BTV xMAP assay and the serotype-specific RT-PCR assays were compared based on quotes provided to The Pirbright Institute from the following suppliers: ThermoFisher, Sigma-Aldrich, and Luminex Corp. Instrumentation costs or staff costs were not considered in the final costing. 

## 3. Results

### 3.1. Assay Optimization

#### 3.1.1. Ratio of Biotinylated:Non Biotinylated Primer

**Figure 1:**
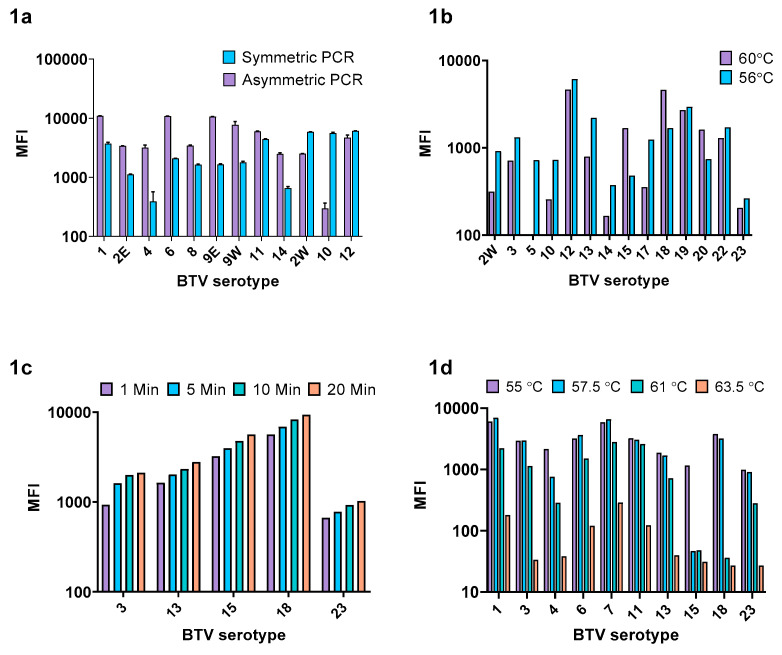
Bluetongue virus (BTV) xMAP assay optimization. Shown are the median fluorescence intensities (MFI) for the four stages of assay optimization: (**a**) symmetric vs. asymmetric PCR, (**b**) PCR annealing temperature, (**c**) SAPE binding duration, and (**d**) capture probe hybridization temperature.

An initial assessment using a subset of serotypes suggested that an asymmetric PCR approach using a 1:4 ratio of non-biotinylated: biotinylated primer would provide the greatest MFI (Figure 1a) and this ratio was subsequently applied to all serotypes. However, western topotype BTV-2w, BTV-10, and BTV-12 were not optimally amplified using this ratio and therefore an equimolar concentration of biotinylated and non-biotinylated primer was chosen for these serotypes.

#### 3.1.2. xMAP PCR Annealing Temperature

The optimal annealing step of the xMAP PCR was investigated at two different temperatures: 60 °C and 56 °C, using a subset of fourteen randomly selected BTV isolates (Figure 1b). Although the average MFI did not differ significantly between the two temperatures (paired t-test, *p* = 0.8), modest improvements in MFI were observed for several of the weakly detected serotypes (such as BTV-2w, BTV-5, BTV-10, and BTV-14) when annealing was performed at 56 °C. Therefore, 56 °C was chosen as the optimal PCR annealing temperature.

#### 3.1.3. Biotin/Microsphere Complex Incubation Temperature and SAPE Incubation Duration

The phycoerythrin signal was found to increase with extended SAPE incubation time for each of the four serotypes tested (Figure 1c). The mean phycoerythrin signal tested across a subset of ten serotypes reduced with each increasing temperature gradient: 55 °C (mean MFI 3112), 57.5 °C (mean MFI 2973), 61 °C (mean MFI 1157), and 63.5 °C (mean MFI 90.5) as shown in Figure 1d. A temperature of 55 °C was chosen as the optimal incubation condition for hybridization of the capture probe to the biotinylated target and 20 min was chosen as the optimal duration for SAPE/biotin binding.

#### 3.1.4. Design and Optimization of the BTV xMAP Serotyping Panels

The initial design of the BTV xMAP assay considered three 9-plex serotyping panels and was designed to minimize the variation in melting temperatures of primers within each panel. This assay was successful in the detection of BTV-1 to BTV-23; however, it was not possible to detect BTV-24 in this format of the assay (Figure S1). A series of duplex assays performed using RT-qPCR demonstrated that amplification of BTV-24 was inhibited in the presence of primers for various other serotypes (Figure S2). When multiple primers were combined in a single reaction, there was a strong relationship between the numbers of primer pairs and the degree of inhibition (*r* = 0.94, *p* ≤ 0.05). Based on these results, the decision was made to redesign the BTV xMAP assay in a five-panel (A–E), 5-plex serotyping format (Table S3).

### 3.2. Detection of BTV Reference Strain Serotypes 1–24

**Figure 2:**
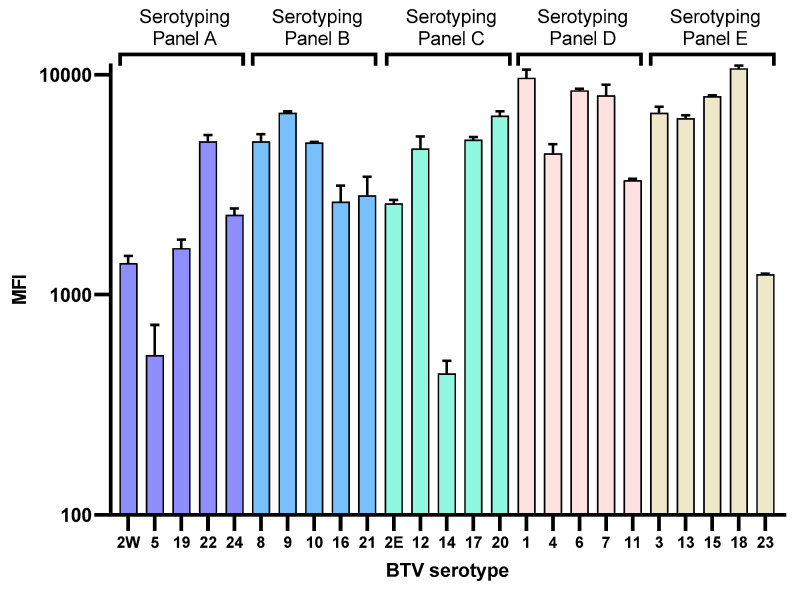
Performance of the BTV xMAP assay to detect BTV reference strains (1–24). For each of the serotyping panels (A–E), the median fluorescent intensity (MFI) is shown on the y axis while the x-axis represents the serotype detected. Samples tested in duplicate; error bars represent standard deviation.

The BTV xMAP assay in 5-plex format was successfully used to detect BTV reference strains 1–24 obtained from the Orbivirus Reference Collection (Figure 2). No off-target cross hybridization between labelled amplicon and microsphere was detected (off-target positive signals with an MFI ≥ 150) (Figure S3). Using the serotype-specific BTV RT-qPCR, C_T_ values of the BTV reference strains ranged from 13.6–28.6 (mean 17.3) as shown in Table 1. Using the BTV xMAP, the MFI ranged from 438.5–10662.3 (mean 4796.3). A moderate correlation was found between C_T_ values and MFI (*r* = −0.54, *p* ≤ 0.05).

### 3.3. Performance of the BTV xMAP Assay on Diagnostic Samples

In addition to the 24 BTV reference strains, a further 79 samples (obtained from clinical samples and proficiency testing schemes) were tested in the evaluation of the BTV xMAP assay. Results from the BTV xMAP assay were compared to those obtained using the group specific and serotype-specific RT-qPCR assays. The BTV group-specific RT-qPCR assay (targeting segment 10) detected BTV in 67 out of 79 samples with C_T_ values ranging between 20.1 and 36.9 (Table S4). The twelve samples that were found to be negative in the group specific RT-qPCR were also found to be negative in the xMAP assay for all twenty-four serotypes tested, resulting in an assay specificity of 100 %. From the 67 BTV-positive samples, the xMAP assay detected a single serotype in 50 samples (Table 2) and multiple serotypes were detected in 9 samples (Table 3). A serotype could not be determined for 8 positive samples by either xMAP or serotype-specific RT-qPCR. In samples containing a single serotype (Table 2) and tested using the serotype-specific RT-qPCR, the C_T_ values ranged from 20.6–36.4 with a mean C_T_ of 28.9. Using the BTV xMAP assay, the MFI for these samples ranged from 183.0–5787.5 with a mean MFI of 2443.0. A number of individual BTV serotypes were detected in the sample set; BTV-1, BTV-2, BTV-3, BTV-4, and BTV-8. In addition, BTV-9, BTV-11, and BTV-15 were identified in samples containing multiple serotypes (Table 3). All samples determined negative in the xMAP were found to be negative in the serotype-specific RT-qPCR.

Using the BTV xMAP assay, multiple BTV serotypes were detected in 9 BTV-positive samples (Table 3). In samples OS 2, OS 6, OS 20, and OS 23, a second serotype was detected using the BTV xMAP assay. In the RT-qPCR, weak amplification for these serotypes was detected but it did not meet the criteria to be considered positive (C_T_ ≤ 38), indicating that the xMAP yielded accurate results. In sample PTS 27, serotype 4 was detected using the xMAP but not using the RT-qPCR. The MFI for this serotype in the xMAP was only slightly above the threshold for positivity, thus, we cannot rule out this being a false positive result. In one sample (OS 7), three serotypes were detected in the serotype-specific RT-qPCR; however, one of these (BTV-4, C_T_ 33.62) was not detected in the xMAP. In comparison with the group-specific RT-qPCR assay, the sensitivity of the serotype-specific RT-qPCR was 79.85% and the sensitivity of the BTV xMAP was 87.55%. A correlation was found between C_T_ values and MFI in the clinical samples (*r* = −0.63, *p* ≤ 0.05).

### 3.4. Limit of Detection

The limit of detection for serotypes BTV-3, BTV-15, and BTV-22 was evaluated using the BTV xMAP assay in comparison with the serotype-specific RT-qPCR assay (Table 4). BTV-3 was detected across a 5-log_10_ dilution, with an average MFI at the lowest dilution of 5902.3 (corresponding C_T_ of 20.1) and an MFI at the highest detectable dilution of 1301.0 (corresponding C_T_ of 33.7). BTV-15 was detected across a 6-log_10_ dilution, with an MFI at the lowest dilution of 7289.0 (corresponding C_T_ of 21.1) and an MFI at the highest dilution of 593.0 (which was beyond the limit of detection in the RT-qPCR). BTV-22 was detected across a 6-log_10_ dilution, with an MFI at the lowest dilution of 2613.3 (corresponding C_T_ of 15.4) and an MFI at the highest dilution of 811.1 (corresponding C_T_ of 34.3). A strong correlation was found between MFI and C_T_ values; however, it was not always found to be significant: BTV-3 (*r* = −0.809, *p* = 0.098), BTV-15 (*r* = −0.995, *p* < 0.001), and BTV-22 (*r* = -0.797, *p* = 0.058). The %CV achieved by the BTV xMAP assay was higher than the RT-qPCR assay and generally increased as the limit of detection was approached. The results obtained for these three BTV serotypes give an approximation of the limit of detection, which may differ depending on which serotype is being detected.

### 3.5. Repeatability

The intraplate and interplate repeatability of the BTV xMAP assay was assessed by testing BTV-positive samples (*n* = 4) in triplicate in a single run (intraplate) and then in triplicate across three different runs (interplate). The mean intraplate %CV was 5.4% and the mean interplate %CV was 13.6%.

### 3.6. Price Comparison with RT-qPCR

A cost comparison was performed to determine if the xMAP could provide a cost-effective approach towards the complete serotyping of BTV serotypes 1–24. To conclusively determine the BTV serotype(s) within a single sample using RT-qPCR, 24 wells of a PCR plate would be required at a total reagent and consumables cost of approximately £36.63. In contrast, using the BTV xMAP assay, a single sample can be tested for BTV-serotypes 1–24 at a cost of approximately £16.51.

## 4. Discussion

BT remains a significant cause of disease in ruminants throughout the world and outbreaks have consequences on animal trade. The development of a high-throughput, multiplex assay for serotyping BTV would provide a cost-effective means of identifying the true picture of BTV epidemiology and may go towards the deployment of appropriate control strategies. We investigated the potential of an xMAP assay for the detection of BTV serotypes, which could provide rapid epidemiological information to facilitate the selection of appropriate vaccines. 

To expedite the development of the BTV xMAP assay, we utilized primer and probes that were originally developed and validated for use in singleplex RT-qPCR assays [34]. In addition, we have used these assays extensively in the OIE Bluetongue reference laboratory for several years to achieve accurate serotyping results. However, the repurposing of these primers still presented a number of challenges, such as overcoming the disparity in primer melting temperatures. An annealing temperature of 60 °C is described in the original publication for the use of these primers [34]. We experimented with an additional annealing temperature of 56 °C which, as recommended in (https://www.ncbi.nlm.nih.gov/pmc/articles/PMC4846334/), is approximately 5 °C lower than the average melting temperature of all primers. We found that a primer annealing temperature of 56 °C achieved the best results, as a higher temperature inhibited the detection of some serotypes at low concentrations. It may be possible to redesign new RT-qPCR assays (utilizing the most recent sequence data) to yield a uniform melting temperature, which could improve the performance of the xMAP assay. Our initial optimization steps identified that a non-biotinylated primer:biotinylated primer ratio of 1:4 provided superior results for most serotypes. Asymmetric PCR has previously been shown to enhance the fluorescent signal strength in downstream xMAP analysis by increasing the abundance of biotinylated cDNA for subsequent hybridization [28,32,36]. Initially, we applied this ratio universally but later found that weakly detected (with a low MFI) serotypes could be enhanced through alteration of the non-biotinylated:biotinylated primer ratio and/or primer concentration. 

The Luminex provided an easily interpretable visualization of the serotype(s) in a sample and has the potential to be used more widely in veterinary diagnostic laboratories. We investigated the repeatability of the BTV xMAP assay and found that the average intraplate variation was lower than 10%, which is considered acceptable for quantitative assays [37]. Although the inter-plate %CV was ~13%, we consider the BTV xMAP as demonstrating acceptable performance criteria to warrant wider dissemination.

Conjugation of the capture probe to the microsphere is a relatively quick and simple procedure to perform. However, this procedure may potentially yield batch to batch variation in the results. Potential differences in the capture probe to microsphere coupling efficiency can be one of the reasons why the xMAP assay is not fully quantitative. We did not attempt to measure the coupling efficiency and therefore could not standardize the MFI between serotypes, and this may explain the variable MFI values recorded between BTV serotypes at high RNA concentrations. A method of standardizing coupling efficiency should be considered by laboratories establishing xMAP assays and employed if a more quantitative assay is required.

Although the MFI varied for each BTV serotype, a lower MFI was not necessarily indicative of poor sensitivity. For instance, the MFI at the lowest dilution of BTV-15, BTV-3, and BTV-22 samples was 7289.0, 5902.3, and 2613.3 respectively. Despite these differences in fluorescence, the BTV xMAP assay was capable of detecting all three serotypes with corresponding RT-qPCR C_T_ values of ~34. A recent manuscript [38] considered previously reported C_T_ values during infection with a single BTV serotype and reported that EDTA blood from BTV infected ovines and bovines during early-stage viremia (3–6 dpi) yielded mean RT-qPCR C_T_ values of 29.7. During peak viremia (7–12 dpi), the mean C_T_ reduced to 25.2. Post peak viremia (13–30 dpi), the mean C_T_ was found to be 28.1. From our work described herein, the investigation of analytical sensitivity (using three serotypes) and diagnostic sensitivity (using 103 samples with a range of different CT values) demonstrate that the BTV xMAP is capable of detecting serotypes at all three stages of infection. However, it is possible that differences in sensitivity may exist between and within BTV serotypes, thus, we can only ascribe an approximate limit of detection that may not be achievable for all serotypes.

The BTV xMAP assay detected multiple BTV serotypes in several samples, which were subsequently confirmed by the serotyping RT-qPCR. Typically, serotype-specific RT-qPCR is performed to determine the dominant serotype (to infer clinical significance) rather than screen samples for all possible serotypes. Although infections with multiple serotypes have been identified in field samples [39,40,41], these may be underreported when using singleplex RT-qPCR detection strategies where samples may only be tested for a limited number of serotypes. Infection of ruminants with multiple BTV serotypes can lead to the emergence of novel, possibly more virulent, BTV strains [42]. Thus, our understanding of BTV epidemiology in areas where multiple serotypes circulate is still limited by the significant costs associated (and time involved) with performing multiple RT-qPCR assays. For instance, the identification of BTV-8 when it first emerged in the Netherlands in 2006 required multiple analyses using the gel-based RT-PCR assays to exclude the BTV serotypes already circulating in Europe (BTV-1, BTV-2, BTV-4, BTV-9, and BTV-16). The identification of BTV-8 could only be made by repeatedly testing for all remaining 19 BTV serotypes. The subsequent development of RT-qPCR assays has allowed for faster analysis times, the limitations of the real-time PCR instruments presenting an impediment to the rapid identification of a new, non-epidemiologically linked BTV serotype. We consider the xMAP assay a novel and appropriate development of these RT-qPCR assays for the rapid determination of BTV serotype.

The cost of running the BTV xMAP assay is substantially less expensive than running multiple singleplex RT-qPCRs, which is approximately twice the cost. We believe that the development of specialized software for xMAP primer(s) and probe(s) could reduce this cost even further in the future and shorten the optimization steps required. The five BTV xMAP serotyping panels (A–E) represent the end result of an exhaustive optimization process which, we consider, will facilitate the implementation of the BTV xMAP assay in other laboratories. Recently, a low-density TaqMan-RT-qPCR array has been described for the classical BTV serotypes [43] and represents an approach to expedite BTV serotype determination. However, this assay utilizes numerous singleplex RT-qPCR assays, allowing for three samples to be fully serotyped on a 96-wellplate, which is less than what can be achieved using the BTV xMAP (*n* = 12). We do not envisage the role of the xMAP BTV assay to be a frontline diagnostic tool for BTV, instead, we foresee the BTV xMAP being well suited to the reanalysis of previously-obtained, known-BTV positive samples from BTV endemic areas (such as African countries) to determine the overall epidemiology of BTV. The BTV xMAP assay accurately detected the BTV serotypes 1–24, but was not designed to detect the atypical BTV serotypes, which do not require notification to OIE [12] and as such were not included in this assay. However, having developed the BTV xMAP assay, we consider it possible and appropriate to create an additional xMAP serotyping panel to incorporate these atypical BTV serotypes and further utilize the capabilities of xMAP technology. 

Overall, we demonstrated that the xMAP Luminex technology provides a cost-effective and robust approach for serotyping BTV. The comparative ease with which BTV serotypes can be screened using the BTV xMAP assay compared to numerous singleplex RT-qPCR assays means that the implementation of this technology will aid in investigating the molecular epidemiology of BTV. 

## Figures and Tables

**Table 1:** Performance of serotype-specific RT-qPCR assay and xMAP assay on BTV reference strains.

Serotyping Panel	ORC ID	Serotype	RT-qPCR C_T_ Value	xMAP MFI
	RSArrrr/02	2W	28.6	1391.3
	RSArrrr/05	5	20.8	574.5
A	RSArrrr/19	19	17.2	1628.8
	RSArrrr/22	22	15.5	4963.0
	RSArrrr/24	24	19.8	2249.4
	RSArrrr/08	8	20.1	4959.0
	RSArrrr/09	9	13.7	6624.0
B	RSArrrr/10	10	17.6	4904.0
	RSArrrr/16	16	20.7	2644.0
	RSArrrr/21	21	14.5	1812.3
	Ind1994/01	2E	14.7	4904.5
	RSArrrr/12	12	14.6	4604.8
C	RSArrrr/14	14	20.5	438.5
	RSArrrr/17	17	15.2	5061.5
	RSArrrr/20	20	16.2	6513.5
	RSArrrr/01	1	15.2	9667.3
	RSArrrr/04	4	15.7	4385.3
D	RSArrrr/06	6	18.9	8483.5
	RSArrrr/07	7	16.4	8038.5
	RSArrrr/11	11	15.4	3313.8
	RSArrrr/03	3	15.5	6701.8
	RSArrrr/13	13	13.6	6326.0
E	RSArrrr/15	15	13.9	7983.8
	RSArrrr/18	18	16.0	10662.3
	RSArrrr/23	23	21.3	1027.0

ORC: Orbivirus Reference Collection ID.

**Table 2:** Performance of serotype-specific RT-qPCR assay and xMAP assay on samples containing a single serotype.

	RT-qPCR	xMAP		RT-qPCR	xMAP		RT-qPCR	xMAP		RT-qPCR	xMAP
Sample (Serotype)	C_T_ Value	MFI	Sample (Serotype)	C_T_ Value	MFI	Sample (Serotype)	C_T_ Value	MFI	Sample (Serotype)	C_T_ Value	MFI
OS 1 (4)	29.6	1038.5	OS 27 (4)	28.6	1120.5	PTS 13 (1)	28.2	4749.0	PTS 32 (N)	N.D.	N.D.
OS 3 (4)	26.5	3321.0	OS 28 (4)	25.6	1621.5	PTS 14 (1)	N.D.	4127.5	PTS 33 (?)	N.D.	N.D.
OS 4 (4)	25.1	3477.0	UKS 1 (N)	N.D.	N.D.	PTS 15 (1)	N.D.	742.0	PTS 34 (?)	N.D.	N.D.
OS 5 (11)	35.9	2934.0	UKS 2 (N)	N.D.	N.D.	PTS 16 (1)	27.9	5258.0	PTS 35 (3)	29.9	1175.8
OS 8 (12)	25.2	260.5	UKS 3 (N)	N.D.	N.D.	PTS 17 (1)	24.1	5787.5	PTS 36 (3)	26.2	1803.3
OS 9 (4)	26.9	2144.0	UKS 4 (N)	N.D.	N.D.	PTS 18 (8)	36.4	183.0	PTS 37 (3)	22.6	2394.3
OS 10 (4)	28.4	2340.0	PTS 1 (1)	27.3	4886.0	PTS 19 (8)	28.2	1318.0	PTS 38 (?)	N.D.	N.D.
OS 11 (4)	23.0	4055.5	PTS 2 (1)	27.5	5074.0	PTS 20 (N)	N.D.	N.D.	PTS 39 (4)	28.8	630.0
OS 12 (4)	27.6	3111.0	PTS 3 (4)	26.3	3878.5	PTS 21 (1)	27.0	4392.0	PTS 40 (?)	N.D.	N.D.
OS 13 (4)	24.0	3942.0	PTS 4 (4)	27.7	3556.0	PTS 22 (1)	34.0	1107.5	PTS 41 (1)	26.2	3142.8
OS 14 (8)	34.4	396.0	PTS 5 (8)	30.9	1935.8	PTS 23 (N)	N.D.	N.D.	PTS 42 (4)	N.D.	1257.5
OS 17 (11)	31.0	2973.5	PTS 6 (8)	33.5	1201.0	PTS 24 (4)	29.5	2415.5	PTS 43 (?)	N.D.	N.D.
OS 18 (4)	30.4	2323.0	PTS 7 (4)	34.9	502.0	PTS 25 (1)	34.9	214.0	PTS 44 (2)	33.6	250.0
OS 19 (?)	N.D.	N.D.	PTS 8 (4)	34.6	262.0	PTS 26 (1)	30.1	3776.5	PTS 45 (1)	34.0	1003.8
OS 21 (?)	N.D.	N.D.	PTS 9 (3)	30.8	1541.3	PTS 28 (N)	N.D.	N.D.	PTS 46 (1)	30.1	2537.8
OS 24 (?)	N.D.	N.D.	PTS 10 (3)	31.1	1714.3	PTS 29 (N)	N.D.	N.D.	PTS 47 (?)	N.D.	N.D.
OS 25 (4)	28.1	977.0	PTS 11 (1)	21.6	5406.0	PTS 30 (N)	N.D.	N.D.	-	-	
OS 26 (4)	20.6	2296.5	PTS 12 (1)	25.1	5595.0	PTS 31 (N)	N.D.	N.D.	-	-	

OS: outbreak sample, UKS: UK-originating samples, PTS: proficiency testing sample, N: negative by group-specific RT-qPCR, N.D.: not detected- C_T_ value > 38 or MFI < 150. ?: positive by group-specific RT-qPCR but no serotype found using serotype-specific RT-qPCR.

**Table 3:** Performance of serotype-specific RT-qPCR assay and xMAP assay on samples containing multiple serotypes.

Sample	Serotypes Detected	RT-qPCR C_T_ Value	xMAP MFI
OS 2	4	28.1	3204.5
	9	N.D.	370.0
OS 6	4	22.9	3864.0
	9	N.D.	200.5
OS 7	9	24.3	3437.5
	11	27.8	3363.0
	4	33.6	N.D.
OS 15	9	29.5	1790.0
	11	31.9	1693.0
OS 16	9	26.5	2989.5
	11	35.3	312.0
OS 20	9	31.6	1681.5
	11	N.D.	248.5
OS 22	9	23.2	3976.5
	11	33.3	1430.5
OS 23	4	21.5	3717.0
	9	N.D.	216.5
PTS 27	15	34.5	556.0
	4	N.D.	164.0

N.D.: not detected- C_T_ value >38 or MFI < 150.

**Table 4:** Analytical sensitivity for the BTV xMAP assay.

	BTV-3 (ZIM2002/03)		BTV-15 (ISR2006/11)		BTV-22 (MAR2005/05)	
Dilution Factor	xMA PMFI (%CV)	RT-qPCR C_T_ (%CV)	xMAP MFI (%CV)	RT-qPCR C_T_ (%CV)	xMAP MFI (%CV)	RT-qPCR C_T_ (%CV)
10^−1^	5902.3 (3.33)	20.1 (0.95)	7289.0 (4.05)	21.1 (0.19)	2613.3 (1.81)	15.5 (0.54)
10^−2^	7050.5 (3.25)	24.1 (2.01)	6496.5 (3.24)	23.7 (0.77)	2767.0 (7.48)	18.5 (0.76)
10^−3^	6912.5 (0.77)	26.9 (0.49)	4629.5 (4.87)	27.4 (0.26)	3051.2 (7.82)	22.1 (1.35)
10^−4^	2919.3 (3.37)	30.2 (0.31)	2721.2 (8.74)	30.8 (0.47)	2797.6 (0.88)	26.0 (0.58)
10^−5^	1301.0 (4.54)	33.7 (0.57)	1554.2 (6.02)	34.5 (1.72)	1801.5 (13.58)	29.6 (0.11)
10^−6^	N.D.	N.D.	593.0 (35.36)	N.D.	811.1 (17.35)	34.3 (1.61)

N.D.: not detected- C_T_ value > 38 or MFI < 150.

## Supplementary Materials

The following are available online at https://www.mdpi.com/2076-2607/8/10/1564/s1, Figure S1: Performance of the BTV 9-plex xMAP assay in detection of BTV reference strains BTV 1–24. Figure S2: Percentage inhibition of BTV-24 amplification in RT-qPCR in the presence of individual (a) or multiple (b) primers for other serotypes. Figure S3: Performance of the BTV 5-plex xMAP assay in detection of BTV reference strains BTV 1–24. Table S1: Primers and capture probes used in the xMAP assay. Table S2: Composition of the BTV serotyping 9-plex panel. Table S3: Composition of the BTV serotyping 5-plex panel. Table S4: Results of samples tested using group specific RT-qPCR BTV assay.
