# Supplementary material for: Simultaneous Detection of Bluetongue Virus Serotypes Using xMAP Technology
Source: Microorganisms. 2020 Oct 11;8(10):1564. doi: 10.3390/microorganisms8101564 (PMC7650804; doi:10.3390/microorganisms8101564)
Supplement: Supplementary file 1 [file microorganisms-08-01564-s001.zip › microorganisms-951626-supplementary.docx]

**
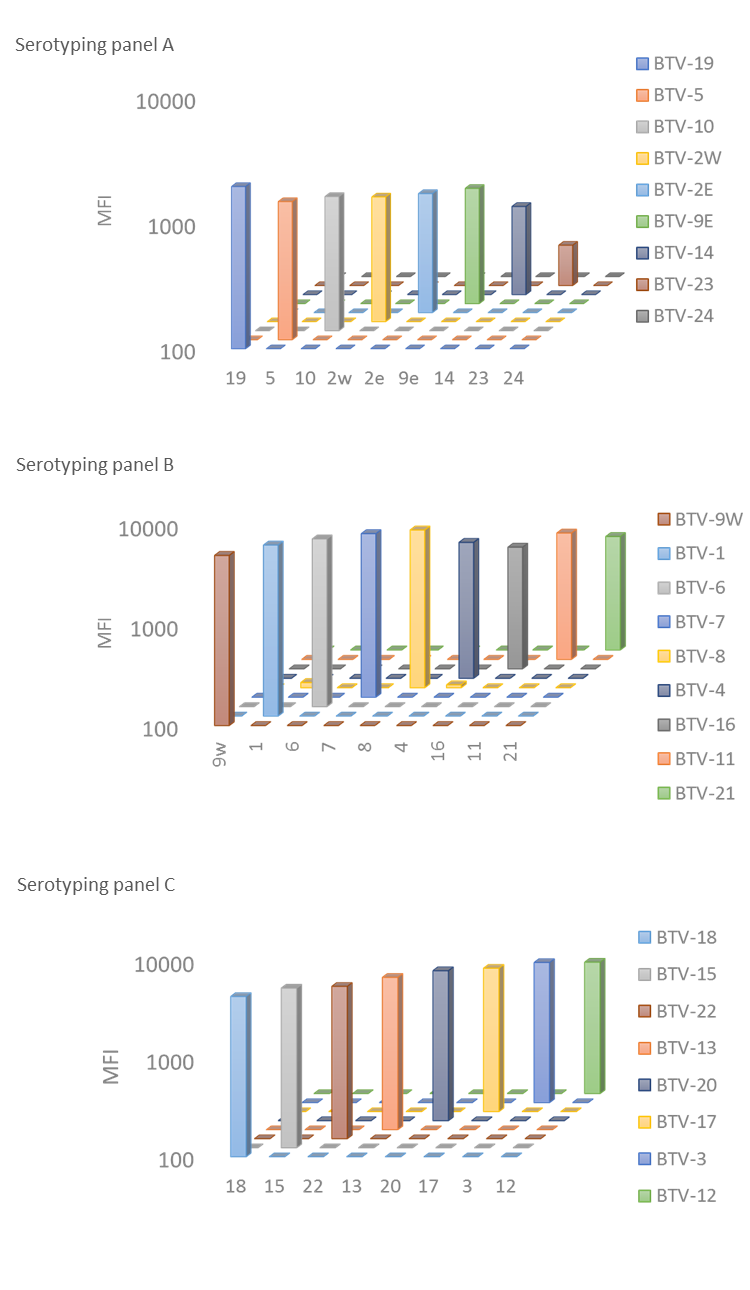
****Figure S1.** Performance of the BTV 9-plex xMAP assay in detection of BTV reference strains BTV 1–24. For each of the serotyping panels (A–C), the median fluorescent intensity (MFI) is shown on the y axis while the x-axis represents the serotype detection channel.

**Figure S2.** Percentage inhibition of BTV-24 amplification in RT-qPCR in the presence of individual (**a**) or multiple (**b**) primers for other serotypes. Sample tested in triplicate. Standard error of the mean is represented by error bars.

**
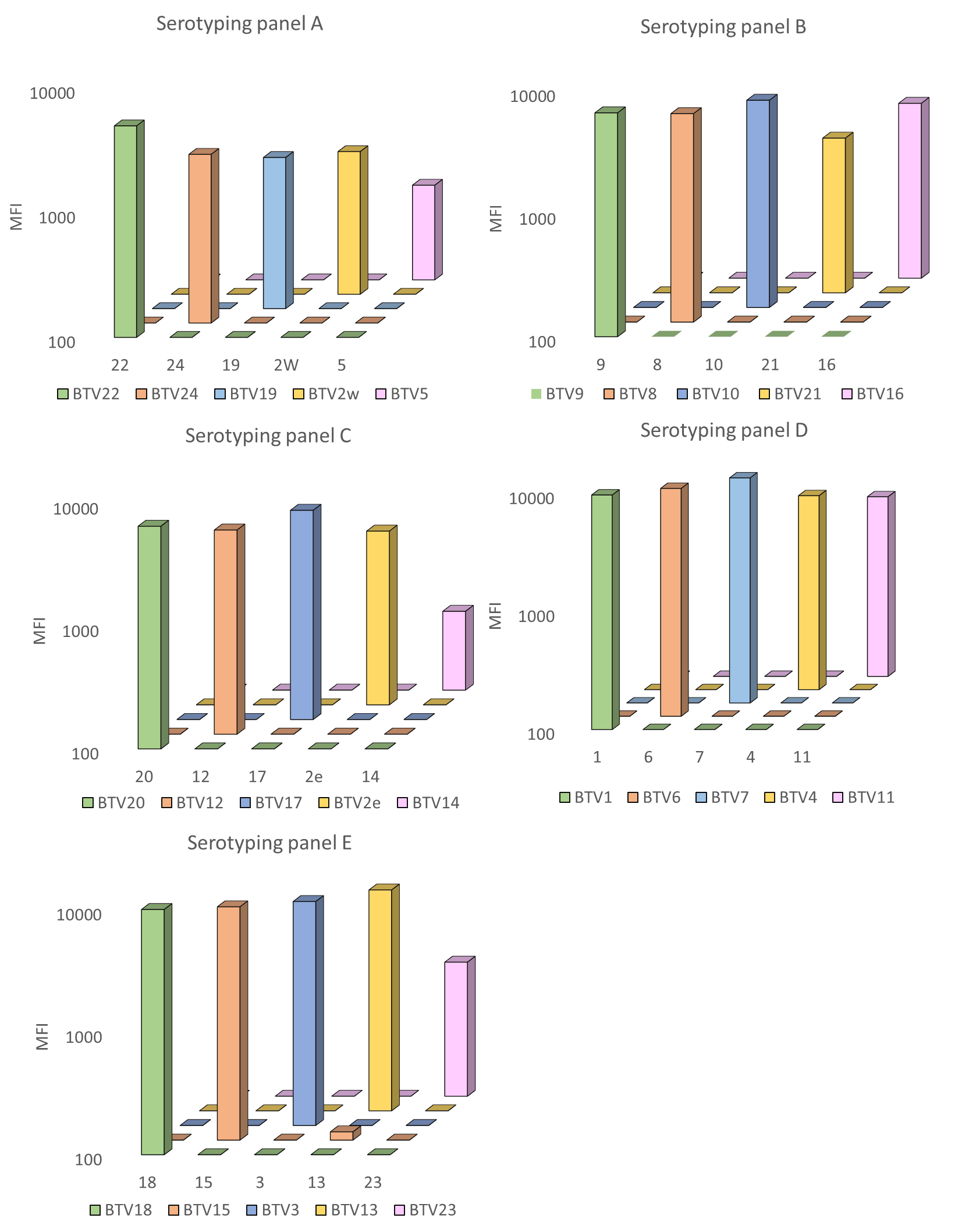
**

**Figure S3.** Performance of the BTV xMAP assay to detect BTV reference strains BTV 1–24. For each of the serotyping panels (A–E), the median fluorescent intensity (MFI) is shown on the y axis while the x-axis represents the serotype detection channel.

**Table S1.** Primers and capture probes used in the xMAP assay

| **Name** | **Primer Sequence (5'-3')** | **Capture probe sequence (5'-3')** |
| --- | --- | --- |
| BTV-1/2575-2597FW | GTATTTCTGAYGGTATTGTYTGG | AmC12-CCGATCACACATCCGAACAAATGC |
| BTV-1/2653-2633RW | Btn-TCATCAGATACCTCGATCGCT |  |
| BTV2_1401-1421FW | Btn-GATGAYRYAARTAYTCTGAG | AmC12-CATTCCATCCACCATCTATAATTTCCCC |
| BTV2_1528-1503RW | GYATCYYTTTCGAARTCRATTGTRAG |  |
| BTV2_60-81FE | GAGCATTTGTTGAAARGTTATG | AmC12-CCAAGATGGCCGACATGACGTATC |
| BTV2_170-148RE | Btn-GATATCRAAYGCGTACATYTCTG |  |
| BTV3/S2/619-640FW | GARCGGTTRTCRACGGAWGARG | AmC12-CYCCRCAGTTTCAYACAATACAGAGGAACCATC |
| BTV3/S2/718-694RW | Btn-TATCRTAAGCGTTATCTCCTARCYG |  |
| BTV4_2470-2488FW | GAACACGAAGATATCGCAG | AmC12-TACCTGTTGTGACRTCCAAGTTGGACAC |
| BTV4_2557-2532RW | Btn-GCATARAGAAGCTARATGTATCTTCA |  |
| BTV-5/S2/08-26FW | GCTTCTCAGGATGGATGAG | AmC12-CCGATWTTKCGRTCGAGCCAAGTTCC |
| BTV-5/S2/101-79RW | Btn-CARRTCRAYCTTAAYRTCRTAYC |  |
| BTV-6/2001-2023F | Btn-GTCGATGTYACACAGTTGATCGT | AmC12-CACCTTGAYTCATCCACACTACGAAC |
| BTV-6/2112-2090R | TAGCACGTCTAATCGTTTCTATG |  |
| BTV7/S2/1608-1631FW | AGTATGTGAGACGTCAATCTCAGA | AmC12-CCACAATCTAGACCCGGCAATATCGC |
| BTV7/S2/1704-1682RW | Btn-AGTATGTGAGACGTCAATCTCAGA |  |
| BTV-8/72-93FW | Btn-GATGGRTATGATTACATCATTG | AmC12-CGGGCTCATCACCTTCCTCTTCAACAC |
| BTV-8/159-138RW | GAATTYCTGTYACATCGTGTCG |  |
| BTV9_1673-1694FW | GGTTATGCTTCAATTACGAACG | AmC12-CTTATATGACACTCGCCCTGCCATC |
| BTV9_1779-1756RW | Btn-GGGTCTTATGTAGGGATGTCTGTG |  |
| BTV9_1706-1724FE | GTATGATACCAGGCCAGCG | AmC12-CAACCCTATCAATGAGACAACGCCAGAC |
| BTV9_1803-1783RE | Btn-GTTCATTTTGAGGATCATCCA |  |
| BTV10/S2/1470-1488FW | TATTRACWACWGAACCAAACCT | AmC12-YCTTGGYNCGCGYTCTGAATTAGTATTYCCRCCY |
| BTV10/S2/1577-1557RW | Btn-GYGARTTRATCCRTTTGTCAT |  |
| BTV11_1510-1530FW | GGATGCGYAYYTGAATATTAG | AmC12-YGTGCTCCCAAGTTATTTCGATCAATGGATCTAC |
| BTV11_1617-1596RW | Btn-ATCTCTCCATGAGTTATTCGCA |  |
| BTV12/S2/999-1019FW | ATACAATYCAGGCYATCMRGA | AmC12- CTCCACCATATGCGCCARCGATAGC |
| BTV12/S2/1136-1116RW | Btn-CAATGATYGTTCCTCGTAAGC |  |
| BTV13/S2/1147-1169FW | Btn-GGTGACGTYTATTATAAATTGCG | AmC12-CTTATATCCCTCACGTACGCTCCAYTCATACC |
| BTV13/S2/1225-1207RW | GGCGATCCARATCYCGWGG |  |
| BTV14/S2/2616-2636FW | GCCATTGARTTTTCTGAYGAYAG | AmC12-CCGGCTTCGCGCGAGRTTYCC |
| BTV14/S2/2758-2734RW | Btn-TCWGTATAYGCCTTAACYGYTCT |  |
| BTV15/S2/29-47FW | Btn-CCTGTGAGCGTGATCGAAC | AmC12-CCCTCCCGATAAAGCGACCATATTCC |
| BTV15/S2/177-156RW | CTTACACCTATGTTTCGCACTC |  |
| BTV16_1221-1243FW | Btn-GCGAGAGCAAGAGAAGTATATCG | AmC12-CCTTCGTTGCTGGCTCTCCCTCTAGATC |
| BTV16_1337-1319RW | GATGTTCGATACGTCTGGG |  |
| BTV16_1193-1213FE | Btn-GACCTGAATATAAACCGCGAG | AmC12-CCTTCGTTGCRGGCTCTCCTTCTAAGTC |
| BTV16_1320-1297RE | ATTAATCAATTCGTACTCCCAGTG |  |
| BTV17/S2/2178-2202FW | TGCTRAAAGAGATCAAATTTGTRCGG | AmC12-CCTCCCTCTGATGTTCCTTGTTCATGATAAC |
| BTV17/S2/2315-2295RW | Btn-ACTTGATCGTATCGTCAAACA |  |
| BTV18/S2/357-381FW | GATTATCAACCACTTAAGGTCGACG | AmC12-CATGTACCATCACGGATAAGCCACGCCC |
| BTV18/S2/451-425RW | Btn-GCTCTCTTTGCGTGTAACCTTACCGTG |  |
| BTV19/S2/2313-2336FW | Btn-AGTGTTGRTATCRCATAAATTACG | AmC12-CCAAACCTATTATARTACGCACCRAGCTCAACC |
| BTV19/S2/2410-2379RW | GGAAAGTYAGATGCGAAATYARRGAAGTCAAT |  |
| BTV20/S2/1838-1856FW | GCAATATGTCCGCATGCTG | AmC12-CCGTAAAACCGCTTTGATGCTGATGGC |
| BTV20/S2/1928-1909RW | Btn-GCTCCGGGCTTAATTTTTCG |  |
| BTV21/S2/1584-1603F | GCCAGATTAAAGATAACGCA | AmC12-CGCTCAACGTAAAGCAGATGACCC |
| BTV21/S2/1686-1669R | Btn-GTAAATCGATAGGGTCCG |  |
| BTV22/S2/1013-1032FW | ATCTCAAGCGGTCAAACAGA | AmC12-CTCCACCAGATACGCCACCGATAAC |
| BTV22/S2/1124-1148RW | Btn-CCATTTCACAYGCTATTATAGTTCC |  |
| BTV23/S2/60-81F | GCGGARYTGTTAGATGGCTATG | AmC12-CGAYGTAAGCACACGYATCGATGAACC |
| BTV23/S2/148-126R | Btn-GGAATTTGWGYRACRTCATGACG |  |
| BTV-24/S2/1901-1919FW | CGAACTAYGAGAAGCTTAYRCC | AmC12-CATCAGACTTACAYGCACCCGAARATAAAY |
| BTV-24/S2/2016-1994RW | Btn-GCGAAAARTCYYTCATATCTA |  |

F; forward primer R; reverse primer, W; Western topotype, E; Eastern topotype BTN; Biotin, Amc12; 5’amino with C12 linker

**Table S2.** Composition of the BTV serotyping 9-plex panel

|  | **Serotype** | **Microsphere set number** | **Primer** | **Final Conc. (µM)** |
| --- | --- | --- | --- | --- |
| Serotyping Panel 1 | 2E | 12 | BTV2 FE | 0.15 |
|  |  |  | BTV2 RE* | 0.6 |
|  | 5 | 13 | BTV5 F | 0.15 |
|  |  |  | BTV5 R* | 0.6 |
|  | 2W | 14 | BTV2 F* | 0.4 |
|  |  |  | BTV2 R | 0.4 |
|  | 10 | 15 | BTV10 F | 0.4 |
|  |  |  | BTV10 R* | 0.4 |
|  | 14 | 18 | BTV14 F | 0.15 |
|  |  |  | BTV14 R* | 0.6 |
|  | 19 | 19 | BTV19 F* | 0.4 |
|  |  |  | BTV19 R | 0.1 |
|  | 9E | 20 | BTV9 FE | 0.15 |
|  |  |  | BTV9 RE* | 0.6 |
|  | 23 | 21 | BTV23 F | 0.15 |
|  |  |  | BTV23 R* | 0.6 |
|  | 24 | 22 | BTV24 F | 0.3 |
|  |  |  | BTV24 R* | 0.6 |
|  | **Serotype** | **Microsphere set number** | **Primer** | **Final Conc. (µM)** |
| Serotyping panel 2 | 4 | 12 | BTV4 F | 0.1 |
|  |  |  | BTV4 R* | 0.4 |
|  | 1 | 13 | BTV1 F | 0.1 |
|  |  |  | BTV1 R* | 0.4 |
|  | 7 | 14 | BTV7 F | 0.1 |
|  |  |  | BTV7 R* | 0.4 |
|  | 6 | 15 | BTV6 F* | 0.4 |
|  |  |  | BTV6 R | 0.1 |
|  | 11 | 18 | BTV11 F | 0.4 |
|  |  |  | BTV11 R* | 0.4 |
|  | 8 | 19 | BTV8 F* | 0.6 |
|  |  |  | BTV 8 R | 0.15 |
|  | 21 | 20 | BTV21 F | 0.1 |
|  |  |  | BTV21 R* | 0.4 |
|  | 9W | 21 | BTV9 FW | 0.1 |
|  |  |  | BTV9 RW* | 0.4 |
|  | 16^†^ | 22^†^ | BTV16 F* | 0.4 |
|  |  |  | BTV16 R | 0.1 |

**Table S2. (c*ontinued*)**

|  | **Serotype** | **Microsphere set number** | **Primer** | **Final Conc. (µM)** |
| --- | --- | --- | --- | --- |
| Serotyping panel 3 | 3 | 12 | BTV3 F | 0.1 |
|  |  |  | BTV3 R* | 0.4 |
|  | 13 | 13 | BTV13 F* | 0.4 |
|  |  |  | BTV13 R | 0.1 |
|  | 15 | 14 | BTV15 F* | 0.4 |
|  |  |  | BTV15 R | 0.1 |
|  | 17 | 15 | BTV17 F | 0.1 |
|  |  |  | BTV17 R* | 0.4 |
|  | 18 | 18 | BTV18 F | 0.1 |
|  |  |  | BTV18 R* | 0.4 |
|  | 12 | 19 | BTV12 F | 0.4 |
|  |  |  | BTV12R* | 0.4 |
|  | 20 | 20 | BTV20 F | 0.1 |
|  |  |  | BTV20 R* | 0.4 |
|  | 22 | 21 | BTV22 F* | 0.4 |
|  |  |  | BTV22 R | 0.1 |

*Biotinylated primer, F: Forward primer, R: reverse primer. E: Eastern topotype, W: Western topotype.

^†^ Primers and microspheres for both Eastern and Western topotype included.

**Table S3.** Composition of the BTV serotyping 5-plex panel

|  | **Serotype** | **Microsphere set number** | **Primer** | **Final Conc. (µM)** |
| --- | --- | --- | --- | --- |
| Serotyping Panel A | 5 | 13 | BTV5 F | 0.15 |
|  |  |  | BTV5 R* | 0.6 |
|  | 2W | 14 | BTV2 F* | 0.4 |
|  |  |  | BTV2 R | 0.4 |
|  | 19 | 19 | BTV19 F* | 0.4 |
|  |  |  | BTV19 R | 0.1 |
|  | 22 | 21 | BTV22 F | 0.1 |
|  |  |  | BTV22 R* | 0.4 |
|  | 24 | 22 | BTV24 F | 0.2 |
|  |  |  | BTV24 R* | 0.8 |
|  | **Serotype** | **Microsphere set number** | **Primer** | **Final Conc. (µM)** |
| Serotyping Panel B | 8 | 19 | BTV8 F* | 0.6 |
|  |  |  | BTV 8 R | 0.15 |
|  | 21 | 20 | BTV21 F | 0.1 |
|  |  |  | BTV21 R* | 0.4 |
|  | 9^†^ | 21 | BTV9 FW | 0.1 |
|  |  |  | BTV9 RW* | 0.4 |
|  | 16^†^ | 22 | BTV16 F* | 0.4 |
|  |  |  | BTV16 R | 0.1 |
|  | 10 | 15 | BTV10 F | 0.4 |
|  |  |  | BTV10 R* | 0.4 |
|  | **Serotype** | **Microsphere set number** | **Primer** | **Final Conc. (µM)** |
| Serotyping Panel C | 12 | 19 | BTV12 F | 0.4 |
|  |  |  | BTV12R* | 0.4 |
|  | 20 | 20 | BTV20 F | 0.1 |
|  |  |  | BTV20 R* | 0.4 |
|  | 17 | 15 | BTV17 F | 0.1 |
|  |  |  | BTV17 R* | 0.4 |
|  | 2E | 12 | BTV2 FE | 0.15 |
|  |  |  | BTV2 RE* | 0.6 |
|  | 14 | 18 | BTV14 F | 0.15 |
|  |  |  | BTV14 R* | 0.6 |

**Table S3. (c*ontinued*)**

|  | **Serotype** | **Microsphere set number** | **Primer** | **Final Conc. (µM)** |
| --- | --- | --- | --- | --- |
| Serotyping Panel D | 4 | 12 | BTV4 F | 0.1 |
|  |  |  | BTV4 R* | 0.4 |
|  | 1 | 13 | BTV1 F | 0.1 |
|  |  |  | BTV1 R* | 0.4 |
|  | 7 | 14 | BTV7 F | 0.1 |
|  |  |  | BTV7 R* | 0.4 |
|  | 6 | 15 | BTV6 F* | 0.4 |
|  |  |  | BTV6 R | 0.1 |
|  | 11 | 18 | BTV11 F | 0.4 |
|  |  |  | BTV11 R* | 0.4 |
|  | **Serotype** | **Microsphere set number** | **Primer** | **Final Conc. (µM)** |
| Serotyping Panel E | 3 | 12 | BTV3 F | 0.1 |
|  |  |  | BTV3 R* | 0.4 |
|  | 13 | 13 | BTV13 F* | 0.4 |
|  |  |  | BTV13 R | 0.1 |
|  | 15 | 14 | BTV15 F* | 0.4 |
|  |  |  | BTV15 R | 0.1 |
|  | 18 | 18 | BTV18 F | 0.1 |
|  |  |  | BTV18 R* | 0.4 |
|  | 23 | 21 | BTV23 F | 0.15 |
|  |  |  | BTV23 R* | 0.6 |

*Biotinylated primer, F: Forward primer, R: reverse primer. E: Eastern topotype, W: Western topotype. ^†^ Primers and microspheres for both Eastern and Western topotype included.

**Table S4.** Results of samples tested using group specific RT-qPCR BTV assay

| Sample | RT-qPCR C_T_ value | Sample | RT-qPCR C_T_ value | Sample | RT-qPCR C_T_ value | Sample | RT-qPCR C_T_ value |
| --- | --- | --- | --- | --- | --- | --- | --- |
| O.S. 1 | 27.0 | O.S. 21 | 32.9 | PTS 9 | 29.5 | PTS 29 | N.D. |
| O.S. 2 | 26.1 | O.S. 22 | 20.8 | PTS 10 | 29.3 | PTS 30 | N.D. |
| O.S. 3 | 26.2 | O.S. 23 | 20.6 | PTS 11 | 21.2 | PTS 31 | N.D. |
| O.S. 4 | 23.9 | O.S. 24 | 29.5 | PTS 12 | 24.6 | PTS 32 | N.D. |
| O.S. 5 | 26.0 | O.S. 25 | 29.2 | PTS 13 | 27.6 | PTS 33 | 30.0 |
| O.S. 6 | 21.7 | O.S. 26 | 22.1 | PTS 14 | 31.7 | PTS 34 | 26.1 |
| O.S. 7 | 21.9 | O.S. 27 | 30.5 | PTS 15 | 35.4 | PTS 35 | 28.6 |
| O.S. 8 | 24.1 | O.S. 28 | 27.0 | PTS 16 | 27.2 | PTS 36 | 24.8 |
| O.S. 9 | 25.5 | UKS 1 | N.D. | PTS 17 | 23.5 | PTS 37 | 20.1 |
| O.S. 10 | 26.8 | UKS 2 | N.D. | PTS 18 | 33.3 | PTS 38 | 22.4 |
| O.S. 11 | 22.4 | UKS 3 | N.D. | PTS 19 | 23.1 | PTS 39 | 31.0 |
| O.S. 12 | 27.6 | UKS 4 | N.D. | PTS 20 | N.D | PTS 40 | 34.9 |
| O.S. 13 | 24.3 | PTS 1 | 26.7 | PTS 21 | 26.5 | PTS 41 | 25.0 |
| O.S. 14 | 28.4 | PTS 2 | 26.5 | PTS 22 | 34.1 | PTS 42 | 27.1 |
| O.S. 15 | 27.7 | PTS 3 | 24.8 | PTS 23 | N.D | PTS 43 | 29.7 |
| O.S. 16 | 25.5 | PTS 4 | 25.5 | PTS 24 | 26.8 | PTS 44 | 26.0 |
| O.S. 17 | 26.4 | PTS 5 | 24.0 | PTS 25 | N.D | PTS 45 | 31.5 |
| O.S. 18 | 30.2 | PTS 6 | 26.4 | PTS 26 | 30.1 | PTS 46 | 28.1 |
| O.S. 19 | 36.9 | PTS 7 | 31.9 | PTS 27 | 32.8 | PTS 47 | 34.4 |
| O.S. 20 | 27.9 | PTS 8 | 32.0 | PTS 28 | N.D. | - | - |

O.S. outbreak sample, UKS, UK-originating samples, PTS, Proficiency testing sample, N.D. Not Detected-C_T_ value ≥ 38
